# Interest in Receiving Nutrition Information Through Social Media Among Food-Security Program Participants in Washington, DC

**DOI:** 10.5888/pcd18.200596

**Published:** 2021-05-20

**Authors:** Adrian Bertrand, Melissa Hawkins, Elizabeth W. Cotter, Donna Banzon, Anastasia Snelling

**Affiliations:** 1 American University, College of Arts and Science, Department of Health Studies, Washington, DC; 2 Martha’s Table, Washington, DC

## Abstract

**Introduction:**

Effective communication approaches are necessary to reach food-security program participants. Accessing food-security programs has been especially challenging during the COVID-19 pandemic. Social media can play an important role in reducing some communication barriers. We examined interest in receiving nutrition information via social media among adults participating in food-security programs in Washington, DC.

**Methods:**

We developed and administered a 22-item survey to adults participating in food-security programs (N = 375). Participants were recruited at Martha’s Table, in Washington, DC, from January through March 2020. We performed bivariate analyses and multinomial logistic regressions to examine predictors of interest in receiving nutrition information via social media.

**Results:**

Sixty-nine percent of participants reported using social media, and 49% expressed interest in receiving nutrition information via social media. Higher levels of self-efficacy and belief in the value of digital technology were associated with greater likelihood of interest in receiving nutrition information via social media (χ^2^_6_ = 139.0; Nagelkerke *R* ^2^ = 0.35; *P* < .001). We found no differences by sex or digital technology access in interest in receiving nutrition information via social media.

**Conclusion:**

Social media is a widely used and a feasible method to reach food-security program participants. Understanding program participants’ interest in receiving health information via social media may help food-security programs plan effective communication strategies to improve food security, especially when in-person participation is limited, such as during the COVID-19 pandemic.

**Summary**

**What is already known on this topic?**

Previous studies evaluated the feasibility of incorporating social media communications into nutrition assistance programs, limiting the focus to age, sex, and barriers to use.

**What is added by this report?**

We demonstrated that characteristics such as self-efficacy and belief in the value of digital technology were closely associated with interest in receiving nutrition information through social media.

**What are the implications for public health practice?**

Our research provides insight into the characteristics of participants who may be responsive to receiving nutrition information through social media.

## Introduction

Food insecurity is a public health problem in the US that has been exacerbated by the COVID-19 pandemic. Food insecurity, defined as “the limited or uncertain availability of nutritionally adequate and safe foods or limited or uncertain ability to acquire acceptable foods in socially acceptable ways” (1), affects more than 11% of US households (2). The number of food-insecure people in the US is estimated to increase because of the pandemic to almost 20%, or 54.3 million Americans (3). In Washington, DC, food insecurity increased from 10.6% in 2018 to 16.0% of the population in 2020 (4).

Differences in access to affordable and nutritious food across socioeconomic status also contribute to health disparities. Food insecurity has health consequences across the lifespan and is associated with increased risk for the development of chronic conditions such as obesity, diabetes, hypertension, coronary artery disease, and asthma (5–8). Furthermore, the pandemic disproportionately affects populations already at risk for food insecurity. In Washington, DC, Wards 7 and 8 account for 22.5% of the city’s population (9), have the highest poverty rates (26.5% and 34.2%, respectively [10]), and are disproportionately affected by food insecurity (11). As of February 2021, residents of Wards 7 and 8 accounted for 35% of the total number of deaths attributable to COVID-19 in Washington, DC (12). During the pandemic, food security assistance programs modified their approaches to serve the community, including finding new locations to provide contactless food distribution (13).

Social media or social networking sites are web pages that allow users to create profiles, share content, and participate in discussions (14) to facilitate communication and community engagement (15). The number of social networking sites users in the US is increasing: 70% of adults use social networking sites today, compared with 5% in 2005 (16). Communication between an organization and its members could be enhanced by using social networking sites. However, access to and use of social networking sites is not necessarily an indication of interest in receiving nutrition information (17).

Studies that explored perceptions of social networking sites among food-security program participants limited their focus to rates of use of social networking sites and barriers to use. A study that examined technology use among participants in the Special Supplemental Nutrition Program for Women, Infants, and Children (WIC) found that 92% of participants had cell phones, yet only 23% accessed social networking sites using their cell phones (18). The study also found differences in the use of social networking sites by age, with “Millennials” (people born from 1982 through 1996, according to the Pew Research Center [19]) more likely to report accessing social networking sites than any other age group (18). In another cross-sectional study, of WIC recipients in remote communities of Alaska, more than 85% of participants reported it was useful to receive nutrition information on cell phones or computers, with email and online videos most preferred (20). Barriers to use of social networking sites for accessing nutrition information included technological problems, lack of access to computers or internet services, high cost, and slow internet connections (20). Furthermore, a 2018 systematic review identified the acquisition of new skills and knowledge by participants as a benefit of using social networking sites in health education programs (21). Exploring feasible, accessible, and innovative approaches to reach and engage participants is critical for food-security programs.

Our project was informed by the widely used Health Belief Model, originally derived from behavioral theory and developed to understand perceived barriers and benefits to adopting disease-prevention strategies (22). Proponents of the Health Belief Model argue that a person’s self-efficacy and perceptions about disease prevention strategies and illness determine the adoption of healthy behaviors (22). In our study, the Health Belief Model offered a structure to discuss and organize findings into recommendations for practice and future research, particularly for increasing the effect of food security and nutrition programs through social networking sites. The objective of our study was to describe interest in receiving nutrition information via social networking sites among adults participating in food-security programs in Washington, DC.

## Methods

We conducted this cross-sectional study from January through March 2020. We obtained institutional review board approval from American University in January 2020.

### Recruitment

Researchers partnered with Martha’s Table, a nonprofit organization that provides nutrition education and assistance to individuals and families in the Washington, DC, area. In 2018, the organization distributed 1.65 million healthy meals. Martha’s Table hosts daily markets from 11 AM to 4 PM; fresh produce and pantry items are free. We recruited study participants from among Martha’s Table market participants by using convenience sampling. Daily from January 14 to February 14, we invited market attendees aged 18 or older to complete a brief survey. We provided a consent form to assenting attendees, after which they completed anonymously a 22-item community social media and nutrition survey. Participants indicated whether they preferred to complete the survey by themselves or to have an interviewer read the survey questions aloud. We gave canvas bags and coloring posters to potential participants as an incentive for participation.

### Data collection

Four graduate students were trained to recruit participants and administer the survey. To ensure fidelity, we provided an interviewer script to all interviewers. Every person waiting to attend the market was invited to participate in the study. A pilot test (n = 73) was conducted at Martha’s Table Market in January 2020 to assess clarity and appropriateness of the survey instrument and strategies to engage market attendees. During the first week of January, 80 shoppers at Martha’s Table’s daily market were invited to participate in the pilot survey, and 73 (91.3%) completed the survey. Of those, 35 (47.9%) completed the survey on their own; 38 participants (52.1%) preferred to have the interviewer read the questions aloud. We made 2 revisions after the pilot test. We added examples of non–social networking websites to the question “Do you use the internet?” to avoid confusing the terms “internet” and “social media,” and we added skip-logic instructions, allowing respondents to skip questions that did not apply to them, based on answers to previous questions.

We invited 424 market attendees to participate; 381 agreed and completed the survey (89.9% response rate). We excluded 6 surveys because the respondents were Martha’s Table employees. The final sample size was 375 surveys, of which most (60.3%, n = 226) were completed with the interviewer reading the questions out loud.

### Measures

The development of the survey instrument was guided by the Health Belief Model (23) and input from leadership at Martha’s Table. The instrument consisted of 3 sections: social media, nutrition, and demographic characteristics (Table 1). We adapted the social media questions from a 2018 Pew Research Center survey (14). The social media section included 13 questions to assess participants’ use of social networking sites, frequency of use, access, and perceptions of the value of social networking sites. The questions in the social media section were used to calculate 3 digital technology subscores: technology use, technology access, and technology value. To calculate the technology use and technology access subscores, we used simple addition; each question had options valued at 0 (no) or 1 (yes). The subscore ranged from 0 to 3 for technology use and from 0 to 2 for technology access. For technology value, each of the 2 questions had options valued at 1 (never), 2 (almost never), 3 (sometimes) to 4 (almost always); the subscore ranged from 1 to 4. All participants were asked if and how they accessed nutrition information. We asked users of social networking sites about topics accessed and their interest in nutrition topics via social networking sites. We assessed participants’ interest in receiving nutrition information via social networking sites, the dependent variable, with 1 question and 3 response options: yes, no, or maybe). We stratified responses by social networking site users and nonusers.

**Table 1:** Instrument Questions, Response Options, and Scores, in a Survey Developed to Assess Interest in Receiving Nutrition Information via Social Networking Sites Among Adults Participating in Food-Security Programs in Washington, DC, January–March 2020

Category	Question	Response Options
**Social media**		
Technology use	1. Do you have a cell phone? 2. Do you use social media pages (ie, Facebook, Twitter, Instagram)? 3. Do you use the internet (ie, visit different sites such as Google, or news sites)?	Yes/no
Technology access	4. Do you access your social media pages using your cell phone? 5. Do you access the internet using your cell phone?	Yes/no
Technology value	6. Do you believe that social media pages give you access to valuable resources? 7. Do you believe that the internet gives you access to valuable resources?	• Never • Almost never • Sometimes • Almost always
Dependent variable	8. Would you be interested in receiving healthy eating information via social media?	• Yes • No • Maybe
Frequency of social media use	9. If you use social media. How often do you typically use it? (check ONE)	• Almost constantly • Several times a day • About once a day • Several times a week • Less than once a week
Open-ended question	10. If you don’t use social media sites. Why? Please explain:	[Write in]
Multiple selection	11. If you use social media. Do you use social media for any of the following?	• Find healthy eating information • Find parenting advice • Find health information • Find information about community events • Do not use social media • Other (please specify)
Multiple selection	12. Do you access healthy eating information using any of the following? (check all that apply)	• Family and friends • Internet websites • Social media • Community Groups • I don’t search for healthy eating information • Other (please specify)
Multiple selection	13. The following is a list of nutrition topics. If you use social media. Please choose 2 that you would like to receive on your social media page.	• Healthy recipes • Healthy grocery shopping tips • Weight loss tips • Tips on how to engage children in healthy eating • Farmers markets calendar • Other (please specify) • Not interested
**Nutrition**		
Nutrition education belief	1. It is important to me to learn about healthy eating. 2. Healthy eating education is an important issue.	• Strongly disagree • Disagree • Agree • Strongly agree
Nutrition self-efficacy	3. How much can you do to help your family and/or friends to engage in healthy eating? 4. How much can you do to help your family and/or friends to value healthy eating? 5. How much can you do to help your family and/or friends to believe they can engage in healthy eating? 6. For parents or guardians: As a parent/guardian, I feel prepared to talk about healthy eating with my child/children.	• Very Little • Little • Some • Very much
**Demographic characteristics**		
Gender	1. Gender (please check one)	• Male • Female • Not listed
Parental status	2. Are you a parent or guardian of a child/children 0 to 18 years of age?	• Yes • No
Age	3. What year were you born? (please write)	[Write in]

The 6 questions in the nutrition section were adapted from a previously validated Teacher Health Survey (24). We used the questions to calculate 2 nutrition subscores, for nutrition education belief and nutrition self-efficacy. For nutrition education belief, response options for the 2 questions were 1 (strongly disagree), 2 (disagree), 3 (agree), and 4 (strongly agree); subscores ranged from 1 to 4. For nutrition self-efficacy, response options to the 4 questions were 1 (very little), 2 (little), 3 (some), and 4 (very much); subscores ranged from 1 to 4. The demographic section asked about age, gender, and parental status. We defined “parent” as a parent or guardian of a child younger than 18 years.

### Data analysis

We performed all statistical analyses using SPSS version 26 (IBM Corp) and set significance at *P* <.05 for all tests. We used descriptive statistics to examine individual items. We examined differences between the mean subscores for the social media and nutrition sections (technology use, technology access, technology value, nutrition education belief, nutrition self-efficacy) by gender and parental status using independent samples *t* tests. To determine reliability, we calculated the Kuder–Richardson Formula 20 (KR-20) for dichotomous measures and Cronbach α for nondichotomous measures. Following the approach of Rammstedt and Beierlein (25), we computed reliability for each subscale score, given that each was a separate construct. The technology use score had a KR-20 reliability of α = 0.66, and technology access had a KR-20 reliability score of α = 0.86. Cronbach α was calculated for technology value score (α = 0.82), nutrition education beliefs (α = 0.92), and nutrition self-efficacy score (α = 0.93). We used χ^2^ tests to analyze the bivariate association between age groups, gender, and parental status and interest in receiving nutrition information delivered via social networking sites. A 1-way analysis of variance (ANOVA) test was performed to examine differences in the mean scores calculated between participants who answered yes, no, and maybe to the question on interest in receiving nutrition information via social networking sites. If the ANOVA revealed significant differences, we used post hoc Tukey multiple comparison tests to determine mean differences between social media and nutrition subscores. We performed multinomial logistic regression to predict the relationship between interest in receiving nutrition information via social networking sites and several gender, age, and social media and nutrition scores.

## Results

Of 375 survey participants who completed the survey, 73.2% were women, and 49.3% were parents. Nearly all participants (98.6%) responded to all survey questions. The average participant age was 53.0 (SD, 16.6). Parents were younger (mean age, 47.2 [SD, 14.4]) than nonparents (mean age, 58.7 [SD, 16.7]) (*t*_373_ = 7.14, *P* < .001). The sample age distribution was consistent with the age distribution of Martha’s Table’s clients. Almost all participants (96.5%, n = 362) used cell phones, and most used social networking sites (69.1%, n = 259) and the internet (80.5%, n = 302). Approximately 49.0% of the participants reported interest in receiving nutrition information via social networking sites, 30% of participants reported no interest, and 21% answered “maybe” to being interested. A total of 198 participants indicated use of social networking sites to find information about community events, 175 participants indicated use of social networking sites to find health information, and 168 indicated use of social networking sites to find nutrition information. 

**Figure 1:**
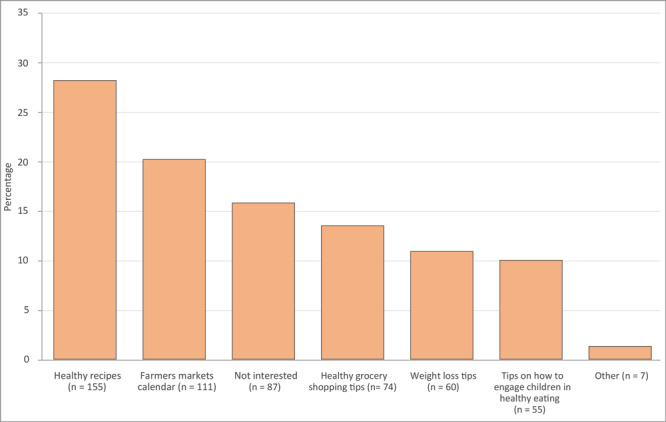
The distribution of survey responses (n = 549) among social media users (n = 259) in study of interest in receiving nutrition information through social media among food-security program participants in Washington, DC, January–March 2020. Survey participants were asked, “Please choose 2 nutrition topics that you would like to receive on your social media page.” Results show the number of responses per topic.

**Figure 2:**
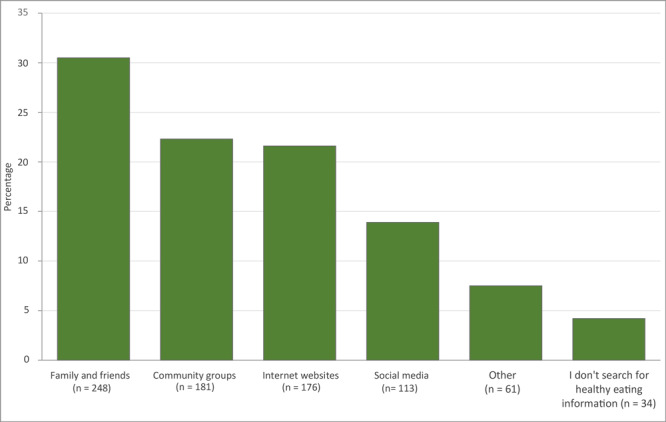
The distribution of survey responses (n = 813) among participants (n = 375) in study of interest in receiving nutrition information through social media among food-security program participants in Washington, DC, January–March 2020. The survey question was, “Do you search for healthy eating information using any of the following?” Participants were asked to check all that applied. Ns are number of responses.

Women and parents had, on average, higher nutrition self-efficacy scores (3.3 and 3.4 respectively) than men and nonparents (3.1 both groups) (Table 2). Survey participants most often chose healthy recipes (n = 155) and farmers market calendars (n = 111) as topics they wanted to receive information on via social networking sites (Figure 1). The most common way to find nutrition information was through family and friends (n = 248) (Figure 2).

**Table 2:** Mean Scores and Demographic Characteristics of Participants in a Survey Developed to Assess Interest in Receiving Nutrition Information via Social Networking Sites Among Adults Participating in Food-Security Programs in Washington, DC, January–March 2020

Category	Score Range	Men		Women		Parent^a^		Nonparent		All	
		n	Mean (SD)	n	Mean (SD)	n	Mean (SD)	n	Mean (SD)	n	Mean (SD)
Technology use	0–3	102	2.4 (0.9)	273	2.4 (0.8)	185	2.6^b^ (0.8)	190	2.3^b^ (0.9)	375	2.4 (0.8)
Technology access	0–2	102	1.2 (0.9)	271	1.3 (0.9)	184	1.5^b^ (0.8)	189	1.1^b^ (0.9)	373	1.3 (0.9)
Technology value	1–4	102	3.0 (0.9)	268	3.1 (0.8)	183	3.2^c^ (0.8)	197	2.9^c^ (0.9)	370	3.1 (0.9)
Nutrition education belief	1–4	102	3.4 (0.8)	273	3.6 (0.7)	185	3.6 (0.7)	190	3.6 (0.7)	375	3.6 (0.7)
Nutrition self-efficacy	1–4	102	3.1^c^ (0.8)	273	3.3^c^ (0.7)	185	3.4^c^ (0.7)	190	3.1^c^ (0.8)	375	3.2 (0.7)

a Defined as a parent or guardian of a child younger than 18 years.

b *P* ≤ .001 between men and women or parents and nonparents.

c *P* < .05 between men and women or parents and nonparents.

**Table:** 

Topic	No. (%) of Responses (n = 549)
Healthy recipes	155 (28.2)
Farmers markets calendar	111 (20.2)
Not interested	87 (15.8)
Healthy grocery shopping tips	74 (13.5)
Weight loss tips	60 (10.9)
Tips on how to engage children in healthy eating	55 (10.0)
Other	7 (1.3)

**Table:** 

Source of Information	No. (%) of Responses (n = 813)
Family and friends	248 (30.5)
Community groups	181 (22.3)
Internet websites	176 (21.6)
Social media	113 (13.9)
Other	61 (7.5)
I don’t search for healthy eating information	34 (4.2)

### Bivariate analyses

We found differences in interest in receiving nutrition information by age group (χ^2^_12_ = 32.0, *P* = .001) and parental status (χ^2^_22_ = 12.5, *P* = .002). The greatest interest was expressed by parents (57.8%) and participants in the group aged 35 to 44 (61.8%). The least interest was expressed by participants aged 75 or older (22%).

In the 1-way ANOVA to examine differences in the mean scores among participants who answered yes, no, and maybe to the question on interest in receiving nutrition information via social networking sites, we found significant differences in the technology use score (*F*_2, 372_ = 79.18, *P* < .001), the technology access score (*F*_2, 370_ = 50.38, *P* < .001), and the technology value score (*F*_2, 367_ = 44.12, *P* < .001). For all 3 scores, a Tukey post hoc test revealed that, on average, participants who responded yes and maybe had higher scores than participants who responded no. We also found significant differences in nutrition self-efficacy scores among the 3 groups (*F*_2, 372_ = 14.51, *P* < .001). A Tukey post hoc test revealed that, on average, participants who responded yes had a higher nutrition self-efficacy score (mean, 3.4) compared with participants who responded no (mean, 3.1) or maybe (mean, 3.0).

### Multinomial logistic regression

We computed 3 multinomial logistic regression models to predict interest in receiving nutrition information via social networking sites. The best fit model included only nutrition self-efficacy, technology use, and technology value scores (χ^2^_6_ = 139.0, *P* < .001, Nagelkerke *R* ^2^ = 0.36) (Table 3). The scores for nutrition self-efficacy and technology use were significant predictors of interest among participants who answered yes, compared with those who answered no (*P* = .004 for nutrition self-efficacy and *P* < .001 for technology use) and those who answered maybe (*P* < .001 for nutrition self-efficacy and *P* = .008 for technology use). The score for technology value was significantly different in a comparison of participants who responded yes and those who responded maybe (*P* < .001). Participants who used digital technology (vs those who did not), highly valued digital technology (vs those who did not), and had high nutrition self-efficacy (vs those who had low nutrition self-efficacy) were more interested in receiving nutrition information through social networking sites.

**Table 3:** Multinomial Logistic Regression of Interest in Receiving Nutrition Information via Social Media Among Adults Participating in Food-Security Programs in Washington, DC, January–March 2020^a^

Independent Variable	B (SE)	Wald χ^2^ (*df*) [*P* Value]	Exp (B) (95% CI)
Comparing participants who answered yes to participants who answered no			
Technology value	−0.59 (0.22)	7.1 (1) [.008]	0.56 (0.36–0.86)
Technology use	−1.21 (0.22)	30.0 (1) [<.001]	0.30 (0.19–0.46)
Nutrition self-efficacy	−0.60 (0.21)	8.2 (1) [.004]	0.55 (0.36–0.83)
Comparing participants who answered yes to participants who answered maybe			
Technology value	−0.60 (0.23)	7.0 (1) [.008]	0.55 (0.35–0.86)
Nutrition self-efficacy	0.75 (0.20)	14.6 (1) [.001]	0.47 (0.32–0.69)

a Survey participant were asked, “Would you be interested in receiving healthy eating information via social media?” Possible answers were yes, no, and maybe.

## Discussion

Our study indicated that social networking sites can provide an efficient and effective way to reach food-security program participants. The importance of access to nutrition information directly relates to healthy food choices and chronic disease prevention (26). Programs to reduce food insecurity and its associated chronic diseases rely on effective communication to support food access. Organizations that provide nutrition assistance in underresourced communities could reach participants with farmers market calendar reminders, 1 of the top 2 nutrition topics on which participants indicated they wanted to receive information via social media.

Our study showed that a high level of nutrition self-efficacy was associated with interest in receiving nutrition information via social networking sites. Participants who were interested in receiving nutrition information via social networking sites, on average, had higher self-efficacy scores than participants who said they were not interested or may be interested. According to Bandura et al (23), self-efficacy is a person’s belief that they possess the ability to succeed in a particular situation. Martha’s Table offers small group programs aimed at helping participants to make healthier decisions. Increasing the availability of these programs could be an effective way to increase nutrition self-efficacy among food-security program participants at Martha’s Table. Previous research determined that age was an important factor in the use of social networking sites (18); however, we found that self-efficacy is a better predictor of interest than age in receiving nutrition information via social networking sites.

Although in our study younger people reported greater use of social networking sites than older participants, the results indicated that use of social networking sites was widely spread among study participants of all ages and is a desired method for receiving nutrition information. A high percentage of participants reported searching for health (43.2%) and nutrition (46.7%) information via social networking sites, and 49% of participants reported interest in receiving nutrition information via social networking sites. In 2014, younger people were the likely users of social networking sites, and the information shared and accessed through social networking sites reflected their interests (17). By 2020, people from a wider range of age groups had become social networking sites users (16); the type of information shared and accessed via social networking sites reflects this increased diversity. Our study found barriers to incorporating social networking sites in food programs, including slow internet speed and the high cost of internet connections, similar to those found by previous researchers (20). It is important that organizations using social networking sites as part of their community outreach efforts are aware of barriers to using these sites.

During the early days of the COVID-19 pandemic, Martha’s Table had to modify its in-person food distribution activities. It increased information sharing via social networking sites, often announcing new locations or formats for food distribution shortly before they happened. COVID-19 caused unrest and forced food assistance organizations to quickly change food delivery strategies; social networking sites helped the organization reach some of their program participants in an equally fast way. Our research suggests that by focusing communication efforts on social networking sites, Martha’s Table was more likely to reach participants with high self-efficacy levels than participants with lower levels of self-efficacy. As the number of users of social networking sites continues to increase in the US, it is essential to further our understanding of how to effectively reach food-security program participants through social networking sites.

Our study has several limitations. One methodologic limitation was that we used a convenience sample. Response bias and social desirability could have occurred given that Martha’s Table programming provides access to nutritious food and our surveys were conducted at their location. The generalizability of our results is limited to food insecure participants in the Martha’s Table’s food assistance program who regularly attend food distribution days. Bias may have resulted from our study sampling approach; program participants who do not regularly attend food distribution days may have different characteristics from participants who regularly attend (ie, the latter engage in more health-seeking behaviors and are more aware of Martha’s Table market offerings). The survey instrument was developed specifically for our study and has not been validated. Furthermore, although the Health Belief Model guided our study, we did not assess the cue-to-action construct, perceived severity construct, or susceptibility construct of the framework. Future studies should examine social factors and other external factors that influence the use of social networking sites and interest in nutrition information. The dependent variable, interest in receiving healthy eating information through social media, could have been interpreted differently by different participants. However, during the pilot period, interviewers did not note any problems in the phrasing of the question or in participants’ understanding of that question. We could not feasibly incorporate cognitive interviewing to identify interpretations of the survey questions among the intended population; such cognitive interviews could be useful in a follow-up study. Our study was cross-sectional; as such, it did not address cause and effect; it provides only a snapshot in time of the population surveyed. It cannot be concluded that interest in receiving information via social networking sites would predict access to and use of the information.

Our findings demonstrate differences between program participants interested in receiving nutrition information through social networking sites beyond age and sex. Characteristics such as self-efficacy and digital technology value are closely associated with interest in receiving nutrition information through social networking sites. In practice, researchers and organizations should consider evaluating several determinants of behavior, such as self-efficacy and digital technology value, when considering the use of social networking sites as a component of their program. Longitudinal studies are needed to determine causation and to examine whether tailoring social media messaging to participants’ interests leads to increased access and interaction with information received via social media. In addition, future studies are needed to examine social networking sites as a method for program participants to receive nutrition information for health promotion using behavior change models, including the Health Belief Model. To our knowledge, our study is the first to explore possible predictors of interest in receiving nutrition information via social networking sites. Our findings can inform researchers and organizations interested in using social networking sites as communication or as a program delivery tool by providing insight into the characteristics of participants who would be responsive to using social networking sites. However, social media is constantly evolving and requires continuous monitoring for research and evaluation.
